# Microglial Exosome miR-7239-3p Promotes Glioma Progression by Regulating Circadian Genes

**DOI:** 10.1007/s12264-020-00626-z

**Published:** 2021-02-02

**Authors:** Xuepei Li, Junwen Guan, Zhou Jiang, Shuting Cheng, Wang Hou, Junjie Yao, Zhengrong Wang

**Affiliations:** 1 Ministry of Health Key Laboratory of Chronobiology, College of Basic Medicine and Forensic Medicine, Sichuan University, Chengdu, 610041 China; 2 Medical Simulation Center, Chengdu First People’s Hospital, Chengdu, 610041 China; 3 Neurosurgery Department, West China Hospital, Sichuan University, Chengdu, 610041 China; 4 Department of Respiratory and Critical Care Medicine, West China Hospital, Sichuan University, Chengdu, 610041 China; 5 Department of Anesthesiology, Wuhan Third Hospital, Tongren Hospital of Wuhan University, Wuhan, 410000 China

**Keywords:** Glioma, Microglia, *Bmal1*, Exosome, miR-7239-3p

## Abstract

Glioma-associated microglial cells, a key component of the tumor microenvironment, play an important role in glioma progression. In this study, the mouse glioma cell line GL261 and the mouse microglia cell line BV2 were chosen. First, circadian gene expression in glioma cells co-cultured with either M1 or M2 microglia was assessed and the exosomes of M2-polarized and unpolarized BV-2 microglia were extracted. Subsequently, we labeled the exosomes with PKH67 and treated GL261 cells with them to investigate the exosome distribution. GL261 cell phenotypes and related protein expression were used to explore the role of M2 microglial exosomes in gliomas. Then a specific miR-7239-3p inhibitor was added to verify miR-7239-3p functions. Finally, the mouse subcutaneous tumorigenic model was used to verify the tumorigenic effect of M2 microglial exosomes *in vivo*. Our results showed that in gliomas co-cultured with M2 microglia, the expression of the BMAL1 protein was decreased (*P* < 0.01), while the expression of the CLOCK protein was increased (*P* < 0.05); opposite results were obtained in gliomas co-cultured with M1 microglia. After treatment with M2 microglial exosomes, the apoptosis of GL261 cells decreased (*P* < 0.001), while the viability, proliferation, and migration of GL261 cells increased. Increased expression of N-cadherin and Vimentin, and decreased E-cadherin expression occurred upon treatment with M2 microglial exosomes. Addition of an miR-7239-3p inhibitor to M2 microglial exosomes reversed these results. In summary, we found that miR-7239-3p in the glioma microenvironment is recruited to glioma cells by exosomes and inhibits *Bmal1* expression. M2 microglial exosomes promote the proliferation and migration of gliomas by regulating tumor-related protein expression and reducing apoptosis.

**Supplementary Information:**

The online version contains supplementary material available at 10.1007/s12264-020-00626-z.

## Introduction

Glioma is the most common primary tumor in the central nervous system (CNS). The prognosis of glioma patients is closely related to the degree of malignancy. Currently, the prognosis of high-grade glioma patients is still not optimistic [1]. Glioma-associated microglial cells (GAMs), a key regulatory component of the tumor microenvironment (TME) in the CNS, play an important role in tumor progression [2]. According to their biological functions, GAMs have been shown to polarize into two distinct phenotypes: M1 microglia that inhibit tumor progression and M2 microglia that function in the opposite way [3–5].

Extracellular microRNAs (miRNAs) can be transmitted stably into the TME by exosomes. Exosome miRNAs, as important mediators of communication in multiple pathological environments, are involved in frequent two-way communication between tumor cells and the TME [6, 7]. Recently, based on the rapid development of biotechnology, especially the high-throughput sequencing, the mechanism of TME regulation has been increasingly investigated. Recent results indicate that, as an important participant in epigenetic regulation, miRNAs are involved in a variety of diseases, including cancers [8–10]. However, due to the complexity of the TME, the interaction between glioma and microglia in the TME, as well as the regulatory mechanism of exosomal miRNAs in specific physiological systems, still need further study.

As one of the most basic physiological regulation systems, the circadian rhythm system, participates in various human biological and physiological activities and maintains internal homeostasis. Therefore, disorder of the circadian rhythm system tends to result in a dysregulated internal environment and causes diseases [11–13]. Due to the complexity and universality of the circadian rhythm system, the study of its molecular mechanism is challenging. Although increasing evidence suggests that circadian dysregulation affects the immune system [14, 15], the exact mechanism of the interaction between miRNAs, clock genes, and glioma progression remains unclear.

In this study, we explored the hypothesis that microglia regulate the expression of glioma clock genes through exosomes, leading to promotion of the proliferation and metastasis of glioma. As a core gene of the circadian rhythm system, *Bmal1* has been associated with the development of multiple types of tumors. Therefore, identifying miRNAs that can regulate *Bmal1* is of great importance for elucidating the molecular mechanism by which M2 microglia promote glioma progression, and to provide a theoretical basis for the development of biologically-targeted therapy for glioma.

## Methods and Materials

### Cell Culture

The mouse glioma cell line GL261 and the mouse microglia cell line BV2 were purchased from the American Type Culture Collection (Manassas, VA, USA). The cell lines were cultured in Dulbecco’s modified Eagle’s medium (Gibco, Waltham, MA, USA) supplemented with 1% penicillin/streptomycin (Thermo Fisher Scientific, Inc.) and 10% fetal bovine serum (Gibco). Cells were cultured at 37°C in a humidified incubator containing 5% CO_2_.

### Preparation of M1/M2 Phenotype BV2 Cell Lines and Co–culture

Interferon (IFN)-γ/lipopolysaccharide (LPS) and Interleukin (IL)-4 were applied to generate the M1 and M2 phenotypes of BV2 cell lines *in vitro*, respectively. LPS and IL-4 were from Abcam (Cambridge, MA, USA). BV2 cells (1 × 10^6^ cells/mL) were seeded into the upper insert of a six-well Transwell plate (Corning Inc., Corning, MA, USA) and cultured at 37°C for 6 h, followed by incubation with either LPS (20 ng/mL) or IL-4 (20 ng/mL) for another 24 h at 37°C. LPS-polarized M1 and IL4-polarized M2 microglia were then washed with phosphate-buffered saline (PBS) and co-cultured with GL261 cells (2 × 10^5^ cells per well) without direct contact for 48 h at 37°C. The co–cultured GL261 cells were then washed and harvested for subsequent experiments.

### Lentiviral Transfection of GL261 Cells with shRNA

*Bmal1* knockdown was performed by RNA interference using lentiviral transfection. Hanbio (Shanghai, China) synthesized lentivirus particles of HBLV-*Arntl*-shRNA-GFP-PURO which expressed short hairpin RNA (shRNA) specific for mouse *Bmal1* (*Bmal1* shRNA) and HBLV-GFP-PURO which expressed green fluorescent protein and purinomycin for negative control (NC). GL261 cells were seeded into 6-well plates with antibiotic-free medium overnight and transfected at 30%–50% confluence with lentivirus at an MOI of 100. At 48 h after transfection, puromycin (5 μg/mL) was used to treat the cells for 7 days, and knockdown efficiency was assessed by Western blot analysis.

### Isolation, Labelling, and Identification of Exosomes

Supernatants were each collected from the BV2 and M2 phenotype BV2 cell culture. Then, exosomes were extracted from the supernatant following the instructions with the ExoQuick-TC kit (Shanran Biotechnology Co., Ltd., Shanghai, China) and suspended in PBS. A transmission electron microscope (TEM; Thermo Fisher Scientific, Waltham, MA, USA) was used to identify the form of the exosomes. The protein content was measured using the BCA protein assay (Thermo Fisher Scientific), and the exosome-specific marker CD9 was detected by Western blot analysis. Fluorescence labeling of exosomes was performed according to a published protocol [16]. Exosomes were labeled with PKH67 Fluorescent Cell Linker Kits (Sigma, USA). PKH67-labeled exosomes were diluted with PBS and ultracentrifuged at 150,000 g and 4°C for 1 h to remove unincorporated dye contamination from exosome labeling reactions. Purified PKH67 exosomes were incubated with GL261 cells and cultured at 37°C for 48 h in a CO_2_ incubator. At the end of incubation, the cells were washed twice with PBS, fixed in 4% paraformaldehyde, and stained with DAPI to visualize nuclei. Cells and the distribution of exosomes were observed under a fluorescence microscope (Olympus, Tokyo, Japan).

### RNA Isolation and Quantitative Real-time PCR (qPCR)

The total RNAs of GL261 cells were purified and enriched with a TRIzol RNA Isolation Kit (Thermo Fisher Scientific). miRNAs were extended by the stem-loop method, and total RNA reverse transcription was performed with the TransScript First-Strand cDNA Synthesis Kit (Thermo Fisher Scientific). After reverse transcription, qPCR was performed to analyze the expression levels of miRNA and mRNA transcripts using specific primers and the QuantiTect SYBR-Green PCR kit (Qiagen, Dusseldorf, Germany). The *β-actin* and *U6* small RNA levels were measured to normalize the mRNA and miRNA levels, respectively. Expression levels were calculated according to the 2^−ΔΔCt^ method. The primers are listed in Table S1.

### Western Blot

Total proteins were extracted from GL261 cells with sodium dodecyl sulfate-polyacrylamide gel electrophoresis (SDS-PAGE) sample buffer (pH 6.8, TrisHCl, SDS, glycerol, 2-mercaptoethanol). Proteins were separated by standard SDS-PAGE using the gel system (Bio-Rad, San Diego, USA) and transferred to polyvinylidene difluoride membranes using a transfer apparatus (Bio-Rad, USA). The membranes were incubated in blocking buffer (5% non-fat milk in TBST buffer) at room temperature for 2 h and then incubated with primary antibodies (1:2000–1:5000) overnight at 4°C. After incubation with secondary antibodies, protein bands were detected by enhanced chemiluminescence according to the manufacturer’s instructions (Image-Quant LAS 4000 mini, Pittsburgh, USA). ImageJ 1.51 (National Institutes of Health, Bethesda, USA) was used for the gray analysis and the relative expression of proteins was normalized to that of GAPDH.

### Cell Counting Kit–8 Assay

One thousand GL261 cells were seeded in a 96-well plate and incubated in complete medium. After culture for 0 h, 24 h, 48 h, 72 h, and 96 h, or treatment with exosomes, cell vitality was measured by the Cell Counting Kit-8 (CCK-8; Dojindo, Tokyo, Japan) assay on the basis of the manufacturer’s instructions.

### Flow Cytometry (FCM)

The apoptosis assay was performed with an Annexin-VFITC/PI apoptosis detection kit (Sigma-Aldrich, St. Louis, MO, USA) following the manufacturer’s manual. After infection or co-culture with M2 phenotype BV2 cells for 48 h, GL261 cells were washed twice with PBS. The cells were re-suspended in 1× binding buffer at 1 × 10^6^ cells/mL, and 5 μL of Annexin-V-FITC conjugate and 10 μL of propidium iodide (PI) were added to each 500 μL cell suspension. Cells were stained by Annexin-V-FITC/PI for 30 min at room temperature. Stained samples were analyzed using a flow cytometer (Partec CyFlow Space, Germany), and the apoptosis rate was evaluated using Cell Quest Pro software (Qume Drive, San Jose, CA, USA).

### Colony Forming Assay

One thousand GL261 cells were plated in 6-well plates after lentiviral transfection or treated with exosomes. Cells were washed twice with PBS after colony formation, then stained with 0.1% crystal violet (Baisibio, Hangzhou, China), and the numbers of colonies per well were counted.

### Transwell Assay

The cell invasion ability was measured by using Transwell chambers (8-μm pore; Coning, USA). GL261 cells (1 × 10^5^ cells/mL) were suspended in serum-free culture medium with or without exosomes and added to the upper Transwell chamber. The lower chamber contained 600 μL of complete medium per well. After 48 h, the non-invading cells on the upper surface were wiped off, and the cells that had invaded to the bottom of the membrane were fixed in methanol and stained with 0.1% crystal violet. Digital images were acquired after air drying and the number of invasive cells was counted under a microscope.

### Wound Healing Assay

GL261 cell migration was measured by wound healing assays. After fixing the Culture Insert (Ibidi, Martin Reid, Germany), GL261 cells (2000 per well) were seeded in 24-well plates and cultured in complete medium with exosomes. After 48 h, the Culture Insert was removed, and the cells were washed with PBS. Cells were then observed under the microscope at 0 h and 12 h. Images of the plates were captured using an inverted microscope (Nikon, Tokyo, Japan) with an attached digital camera and the scratch areas were quantitated with ImageJ software. The percentage of wound healing was used as an observational indicator. Percentage wound healing = (0 h scratch area −12 h scratch area)/0 h scratch area.

### Animal Studies

A total of 18 nude mice (8-week-old males, weighing 22 ± 2 g) were provided by the Sichuan University Laboratory Animal Center (Sichuan, China). The mice were fed in a humidified room (55% ± 10%) at 21 ± 2°C with a cycle of 12 h light/12 h dark. Cells from the mouse glioma line GL261 in the log phase were implanted subcutaneously into nude mice. After 7 days, three groups of mice were intravenously injected with PBS or equal volumes of BV2 or BV2 (M2) exosomes through the tail vein. After the injection, tumor length and diameter, as well as the body weight of each nude mouse were recorded every 3 days. Tumor volume was calculated as (length × diameter^2^)/2. After the mouse was killed, tumor tissue was carefully isolated. Part of the tumor tissue was fixed in 4% paraformaldehyde and then processed for paraffin embedding. The remaining fresh tumor tissue was used to test the relative expression levels of *Bmal1* and miR-7239-3p by RT-qPCR. All animal care protocols and experimental procedures were approved by Institutional Animal Care and Use Commitee of Sichuan University.

### Histopathological and Immunohistochemical Analyses

Tumor tissue embedded in paraffin was cut into 5-μm sections and stained with hematoxylin and eosin for histomorphometry. In the immunohistochemistry (IHC) assay, tumor tissue was stained with debranching enzyme-conjugated anti-Bmal1 (Abcam, USA). Fluorescence intensity was quantified from at least five sections. All samples were observed using an inverted microscope (Eclipse Ti, Nikon, Tokyo, Japan).

### TUNEL Assay

To investigate tumor tissue apoptosis, the terminal deoxynucleotidyl transferase-mediated dUTP nick end-labeling (TUNEL) assay was performed using a cell death detection kit (Roche, San Francisco, USA). In detail, tumor tissue sections were deparaffinized and rehydrated, and then maintained in the TUNEL reaction mixture for 60 min and Converter-POD solution for 30 min in the dark at 37°C. Subsequently, the samples were exposed to diaminobenzidine substrate for 6 min and observed under the Leica microscope. The apoptosis index was used to evaluate the apoptosis of cells in each group [apoptosis index = (number of apoptotic cells / total number of cells) × 100%].

### Statistical Analysis

All of the statistical analyses were carried out using GraphPad Prism 6. Values are presented as mean ± SD with at least three independent experiments in triplicate. Student’s *t* test was used for comparison of data. One-way ANOVA was used for comparison of means between multiple groups, and Pearson’s test was used for correlation analysis of mRNAs. The statistical significance parameter was set at *P* < 0.05.

## Results

### M1/M2 Phenotype Microglia Regulate Circadian Gene Expression of Glioma

**Fig. 1:**
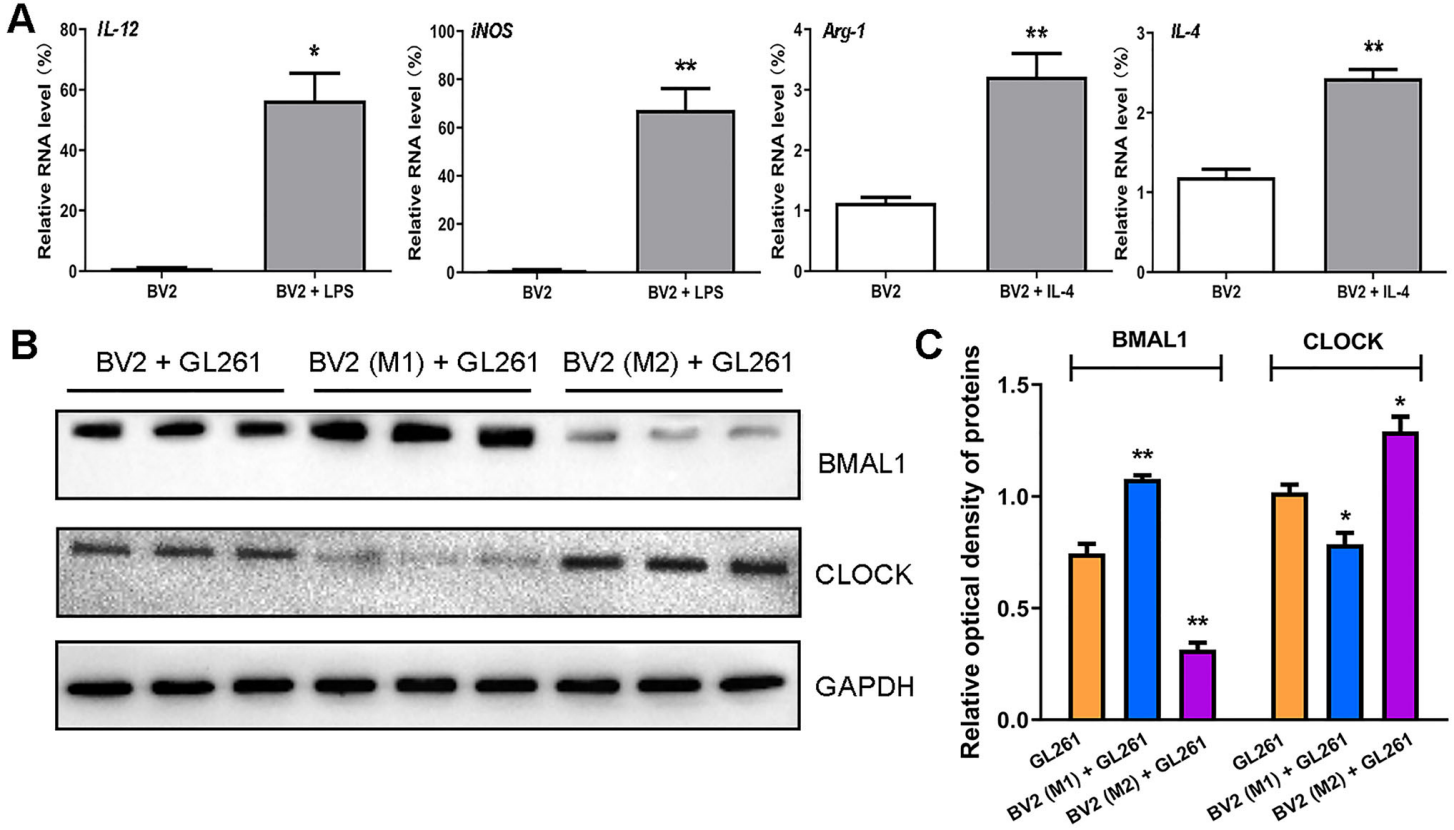
M1/M2 phenotype microglial exosomes regulate circadian gene expression of glioma. **A** Real-time qPCR was used to verify the expression of microglia markers in BV2 cells. The internal reference gene was *β-actin.* **B** Western blot was used to verify the expression of two core clock genes in three groups of GL261 cells. **C** The results of optical density analysis (*n* = 3). The internal reference protein is GAPDH. *P < 0.05, **P < 0.01.

LPS and IL-4 were used to induce BV2 to the M1 and M2 phenotype, respectively. After 24 h, the expression of microglial markers was assessed by qPCR. The results showed that, in LPS-treated BV2 cells, the expression of M1 phenotype markers *IL-12* (*P* < 0.05) and *iNOS* (*P* < 0.01) were increased, and in IL-4-treated BV2 cells, the expression of M2 phenotype markers *Arg-1* (*P* < 0.01) and *IL-4* (*P* < 0.01) were increased (Fig. 1A). This result indicates successful induction of the M1 and M2 phenotype.

M1/M2 phenotype BV2 cells were co-cultured with GL261 glioma cells for 48 h. The protein expression of the circadian genes *Clock* and *Bmal1* in GL261 cells was assessed by Western blot. The results showed that the expression level of BMAL1 protein was increased (*P* < 0.01) in the BV2 (M1) + GL261 group, but significantly reduced (*P* < 0.01) in the BV2 (M2) + GL261 group. On the contrary, expression the of CLOCK protein was reduced (*P* < 0.05) in the BV2 (M1) + GL261 group, but amplified (*P* < 0.05) in the BV2 (M2) + GL261 group (Fig. 1B, C).

### M2 Phenotype Microglia Promote Glioma Proliferation

**Fig. 2:**
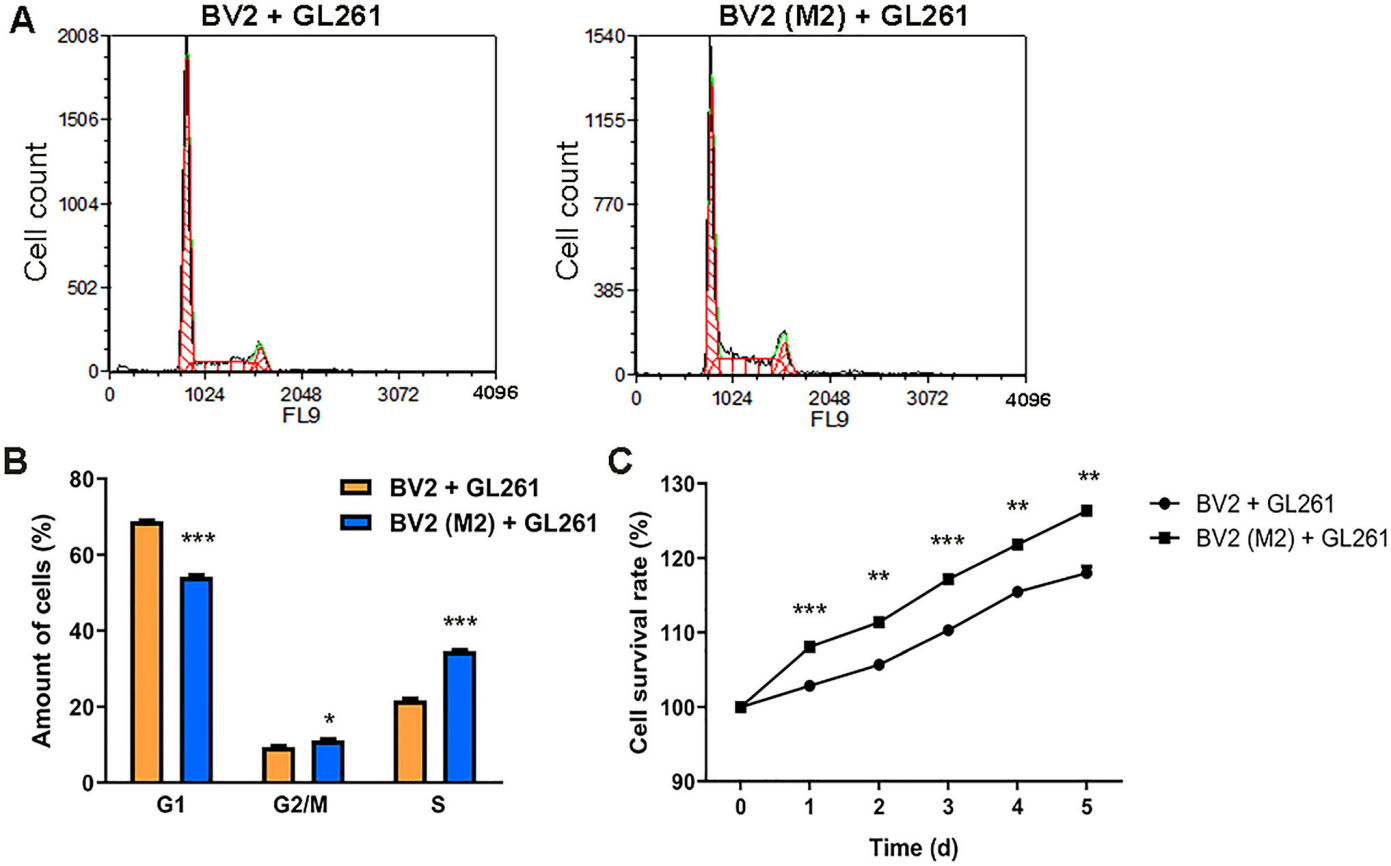
M2 phenotype microglia promote glioma proliferation. **A** Cell cycle of GL261 was detected by flow cytometry (FCM) following PI staining. The abscissa represents the DNA content and the ordinate represents the cell counts. **B** The results of cell cycle analysis. Yellow and blue bars represent the GL261 + BV2 group (*n* = 3) and the GL261 + BV2 (M2) group (*n* = 3), respectively. **C** Survival curve of GL261 cells within 1–5 days after co-culture (CCK-8 assay). The abscissa is the number of days and the ordinate is the cell survival rate (*n* = 3). *P < 0.05, **P < 0.01, ***P < 0.001.

M2 phenotype BV2 cells were co-cultured with GL261 glioma cells and their cell cycle was measured by PI staining after 48 h. The results showed that the population of the GL261 + BV2 (M2) group in the G1 phase was lower than that of the GL261 + BV2 group (*P* < 0.01), while the population in the G2/M and S phases was higher than that of the GL261 + BV2 group (*P* < 0.05, *P* < 0.001; Fig. 2A, B). The survival rate of glioma cells was continuously measured for 5 consecutive days and this showed that the survival rate in the GL261 + BV2 (M2) group was higher than that in the GL261 + BV2 group (*P* < 0.01, *P* < 0.001; Fig. 2C). These results suggest that M2 phenotype microglia improve the survival of glioma cells and promote their proliferation.

### Down-regulation of Bmal1 Promotes Proliferation and Migration of GL261 Cells

**Fig. 3:**
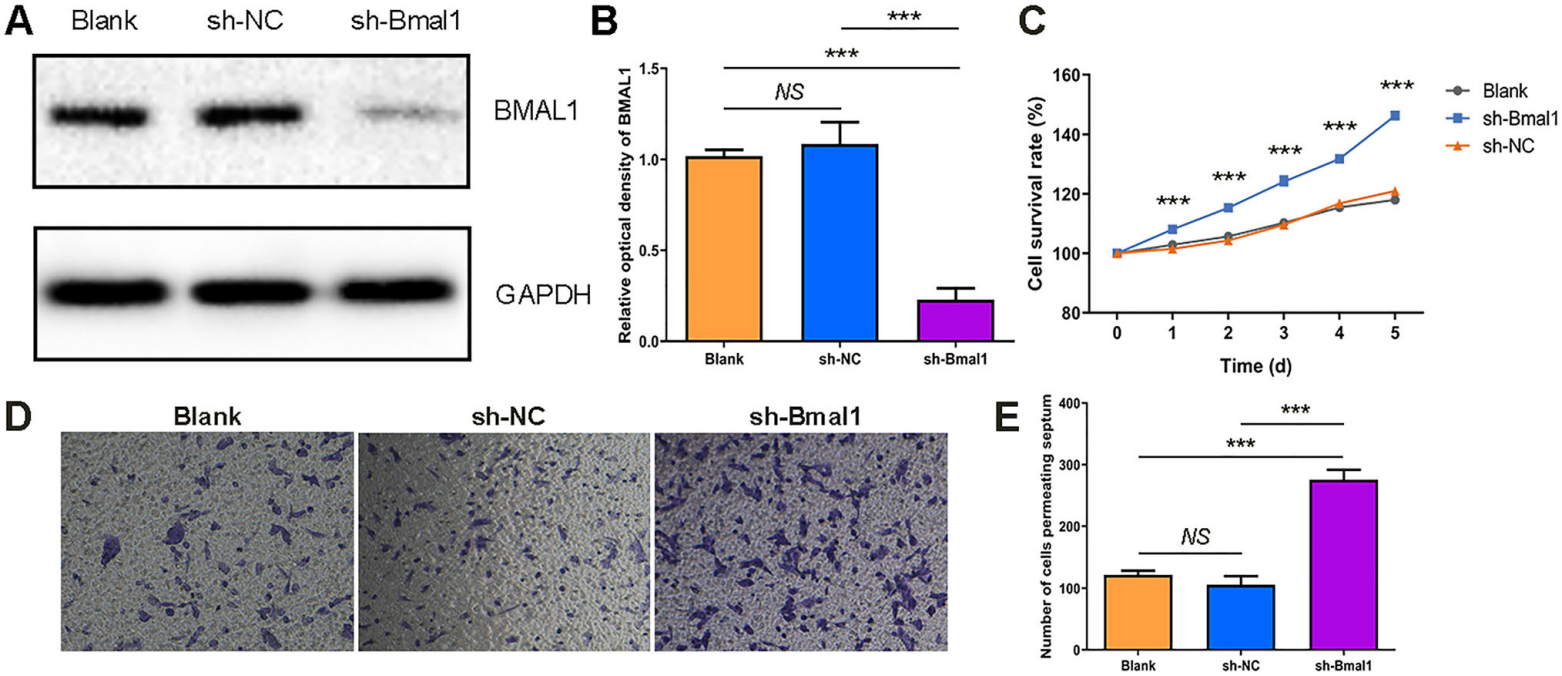
Establishment of *Bmal1* down-regulation in GL261 cells and its effect on glioma proliferation. **A** Western blot was used to detect the differences in the expression levels of BMAL1 protein in three groups of cells. Blank was the blank control group, sh-NC was the negative control group transfected with HBLV-GFP-PURO, sh*-Bmal1* was the study group transfected with the *Bmal1* interfering virus HBLV-*Arntl*-shRNA-GFP-PURO. **B** The result of optical density analysis. The internal reference protein is GAPDH (*n* = 3). **C** Survival curve of GL261 cells (CCK-8 assay). The abscissa is the number of days and the ordinate represents cell survival rate. (*n* = 5). **D** Microscopic images of GL261 cells penetrating the chamber after crystal violet staining (× 100, *n* = 3). **E** The formed clones of GL261 cells after crystal violet staining (*n* = 3). ***P < 0.001.

We next knocked down endogenous *Bmal1* using a lentiviral shRNA system. The effect of lentiviral interfering sequences was assessed by comparing the expression levels of BMAL1 protein in three groups of cells: the *Bmal1* knockdown group (sh-*Bmal1*) transfected with HBLV-*Arntl*-shRNA-GFP-PURO, the blank control group (Blank), and the negative control group (sh-NC) transfected with HBLV-GFP-PURO. The results showed that BMAL1 protein expression was reduced in the sh-*Bmal1* group (*P* < 0.05), and there was no difference in expression between the Blank and sh-NC groups (Fig. 3A, B). These results suggest that we successfully integrated the interference sequence of *Bmal1* into the genome of GL261 cells.

We evaluated *Bmal1*-knockdown cells by CCK-8 assay and transwell migration. The CCK-8 results demonstrated that the survival rate in the sh-*Bmal1* group was higher than that in control groups (*P* < 0.001; Fig. 3C). In addition, the results of the transwell assay showed an increase in the number of cells crossing the diaphragm in the sh*-Bmal1* group (*P* < 0.05; Fig. 3D, E). Taken together, down-regulation of the *Bmal1* gene promotes the proliferation and migration of GL261 cells *in vitro*.

### miR-7239-3p is Up-regulated in M2 Phenotype Microglia Exosomes

**Fig. 4:**
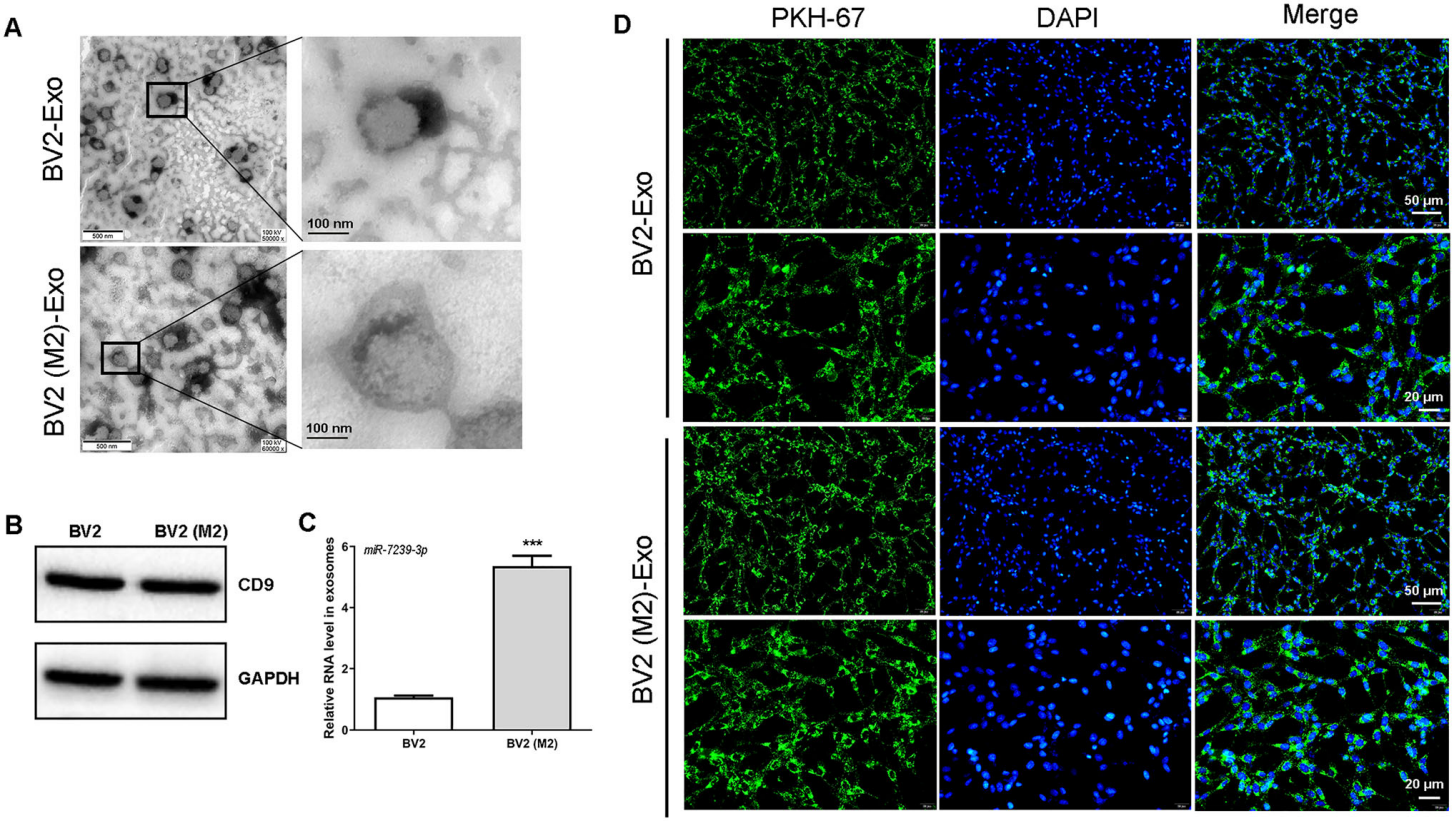
Distribution and identification of exosomes and expression of miR-7239-3p. **A** Transmission electron microscopy imaging of M2 microglia-derived exosomes. Scale bar, 500 nm (left panels); Scale bar, 100 nm (right panels). The enlarged picture shows the structure of the exosomes. Scale bar, 100 nm (*n* = 3). **B** Expression of exosomal markers CD9 in BV2 (M2) exosomes and BV2 exosomes (*n* = 3). **C** Real-time qPCR verified the expression of exosome miR-7239-3p in two groups of microglia cells. The internal reference gene was *U6*. ***P < 0.001 (*n* = 3). **D** The localization of exosomes in GL261 cells was observed by fluorescence microscope. DAPI for nuclear staining, blue fluorescence; PKH-67 for exosome labeling, green fluorescence. Scale bar, 50 μm (the first, and third rows); Scale bar, 20 μm (the second, and fourth rows) (*n* = 3).

Next, exosomes derived from M2-polarized and unpolarized BV-2 microglia were identified using TEM and Western blot analysis. As shown in Figure 4A, the typical cup-shaped membrane vesicle morphology was observed. Western blot analysis confirmed the expression of the exosome marker CD9 (Fig. 4B), indicating that microglial exosomes were successfully isolated. TargetScan that targets the *Bmal1* 3’UTR, and miR-7239-3p was selected to predict miRNAs. RT-qPCR was used to quantitate the miR-7239-3p expression level in exosomes. The results showed that the expression of exosomal miR-7239-3p in M2 microglia was significantly up-regulated (Fig. 4C).

To explore how microglia-derived exosomes interact with gliomas, we labeled exosomes with PKH-67 and observed their distribution in gliomas. The results showed that exosomes were evenly distributed in the cytoplasm of glioma cells after incubation for 12 h (Fig. 4D). This suggests that glioma cells are able to take up microglial exosomes, likely by endocytosis. Moreover, this result also provides evidence for the correlation between up-regulated miR-7239-3p expression in glioma cells and the presence of M2 microglial exosomes.

### M2 Microglial Exosomes Promote Glioma Progression

**Fig. 5:**
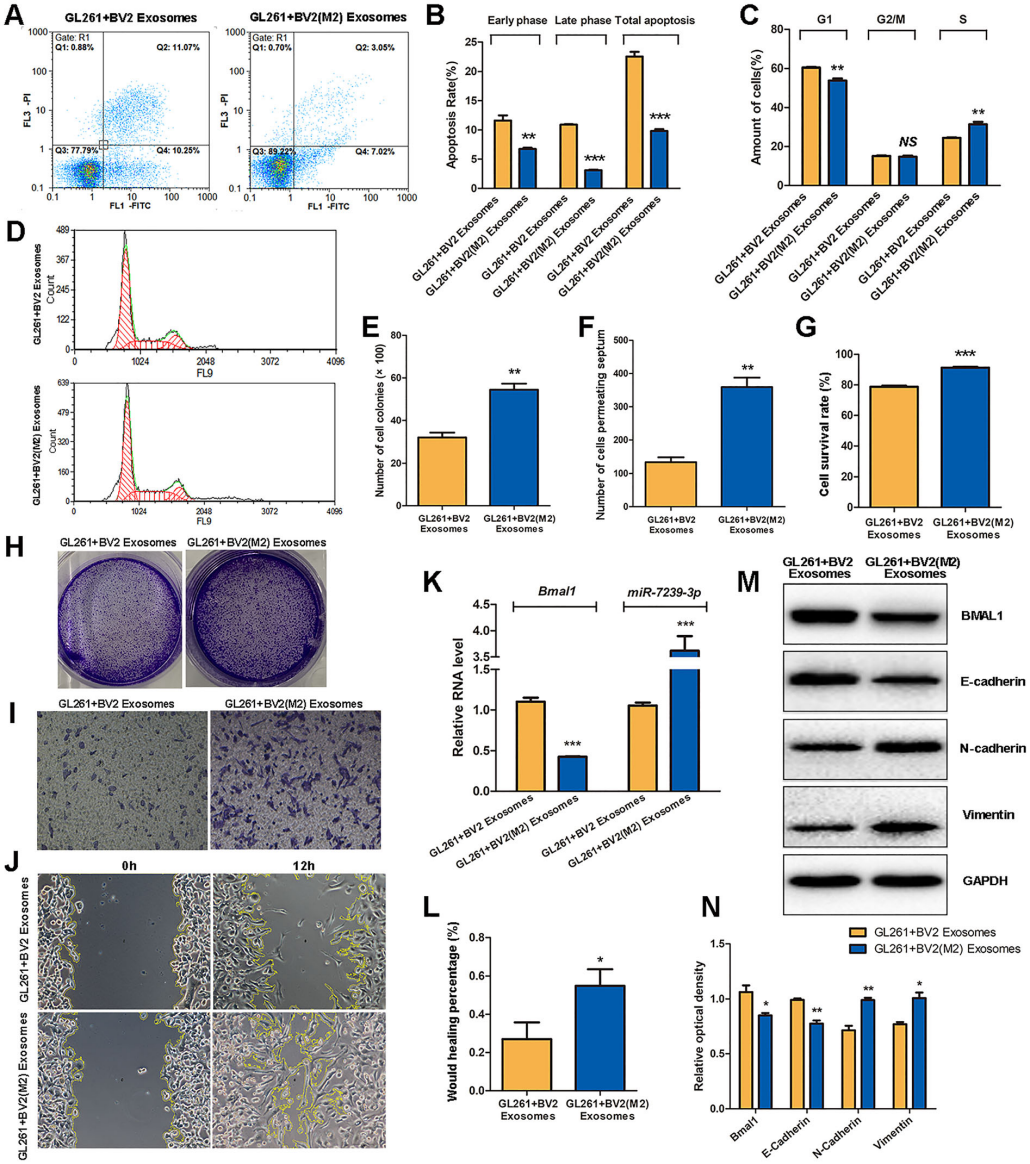
M2 phenotype microglial exosomes promote glioma progression. **A** Cell apoptosis and cell cycle of GL261 was detected by FCM following PI staining. The abscissa represents Annexin V, and the ordinate represents PI. **B** FCM results of Apoptosis. **C, D** FCM results of cell cycle. The abscissa represents the DNA content and the ordinate represents the number of effective cells. **E** The statistical results of the colony number in each group. **F** The statistical result of the number of cells penetrating the chamber in each group. **G** The statistical results of CCK8 assay for cell survival. **H** The formed clones of GL261 cells after crystal violet staining. **I** The microscopic images of GL261 cells penetrating the chamber after crystal violet staining (× 100). **J** The microscopic images of GL261 cell scratch experiments. **K** The relative expression levels of *Bmal1* and miR-7239-3p RNA in the two groups were detected by real-time qPCR. The internal reference protein is β-actin. **L** The statistical results of cell scratch experiments. The vertical axis represents the wound healing percentage. **M** Protein bands of relevant proteins in the two groups were detected by Western blot. **N** Quantitative analysis of protein expressions (*n* = 3). *P < 0.05, **P < 0.01, ***P < 0.001.

After glioma cells were incubated with either unpolarized BV2 exosomes or M2 microglial exosomes, the apoptosis and cell cycle of GL261 cells were assessed by flow cytometry. The results showed that in the GL261 + BV2 (M2) Exosomes group, the early apoptosis, late apoptosis, and total apoptosis were reduced (*P* < 0.01, *P* < 0.001, *P* < 0.001; Fig. 5A, B) and the population of cells in the G1 phase was also reduced (*P* < 0.01), while the S phase population was increased (*P* < 0.01; Fig. 5C, D).

Subsequently, colony-forming and CCK8 assays were used to measure the cell proliferation, and transwell and wound-healing assays were used to evaluate invasion and migration, respectively. The results showed that the GL261 + BV2 (M2) Exosomes group displayed and more colonies (*P* < 0.01; Fig. 5E, H), and showed an increase in the number of cells crossing the diaphragm (*P* < 0.01; Fig. 5F, I). The cell survival rate in the GL261 + BV2 (M2) Exosomes group was higher (*P* < 0.001; Fig. 5G). In addition, the percentage wound healing of the GL261 + BV2 (M2) Exosomes group was higher than that of the GL261 + BV2 Exosomes group (*P* < 0.05; Fig. 5J, L).

RT-qPCR was used to quantitate the RNA expression of *Bmal1* and miR-7239-3p *in vitro*, and Western blot was used to assess the expression of tumor-related proteins. The RT-qPCR results showed that the expression of *Bmal1* in the GL261 + BV2 (M2) Exosomes group was reduced (*P* < 0.001), while the expression of miR-7239-3p was increased (*P* < 0.001; Fig. 5K). Western blot analysis showed that the expression of BMAL1 and E-Cadherin were reduced in the GL261 + BV2 (M2) Exosomes group (*P* < 0.05, *P* < 0.01), while the expression of N-Cadherin and Vimentin were increased (*P* < 0.05, *P* < 0.01; Fig. 5M, N).

### miR-7239-3p Inhibitor Reverses the Tumor-promoting Effects of M2 Phenotype Microglia Exosomes

**Fig. 6:**
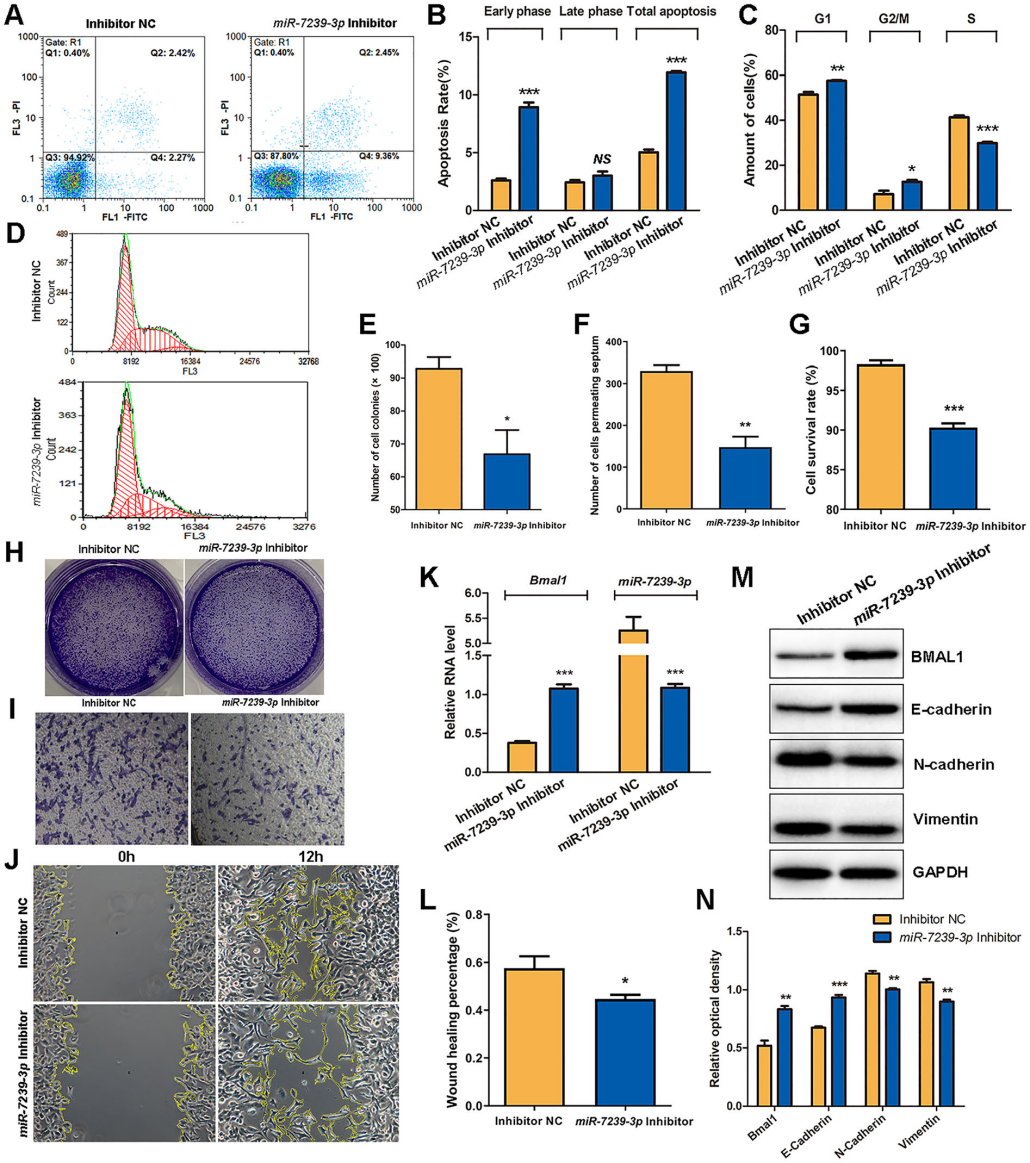
miR-7239-3p inhibitor reversed the tumor-promoting effects of M2 phenotype microglial exosomes. **A** Cell apoptosis and cell cycle of GL261 were detected by FCM following PI staining. The abscissa represents Annexin V, and the ordinate represents PI. **B** FCM results of Apoptosis. **C, D** FCM results of cell cycle. The abscissa of D represents the DNA content and the ordinate represents the number of effective cells. **E** The statistical results of the colony number in each group. **F** The statistical result of the number of cells penetrating the chamber in each group. **G** The statistical results of CCK8 assay for cell survival. **H** The formed clones of GL261 cells after crystal violet staining. **I** The microscopic image of GL261 cells penetrating the chamber after crystal violet staining (×100). **J** The microscopic image of GL261 cell scratch experiments. **K** The relative expression levels of *Bmal1* and miR-7239-3p RNA in the two groups were detected by real-time qPCR. The internal reference protein is *β-actin*. **L** The statistical results of cell scratch experiments. The vertical axis represents the wound healing percentage. **M** Protein bands of relevant proteins in the two groups were detected by Western blot. **N** Quantitative analysis of protein expressions (*n* = 3). *P < 0.05, **P < 0.01, ***P < 0.001.

In order to establish that miR-7239-3p is an important intermediate molecule in the promotion of glioma progression by M2 microglial exosomes, we examined the effects of an miR-7239-3p inhibitor and miR-7239-3p inhibitor negative control (inhibitor NC) on glioma cells by measuring the growth of GL261 cells and their expression of tumor-related proteins. The results of flow cytometry showed that the population of GL261 cells in early apoptosis and total apoptosis were increased (*P* < 0.001) in the miR-7239-3p Inhibitor group (Fig. 6A, B), and the number of GL261 cells in the G1 and G2/M phases of this group increased (*P* < 0.05, *P* < 0.01), while the number of cells in S phase was reduced (*P* < 0.001; Fig. 6C, D). These results suggest that miR-7239-3p accelerates glioma proliferation and reduces glioma apoptosis. Intriguingly, miR-7239-3p mainly affected the early apoptosis of GL261 cells, but had little effect on late apoptosis.

The results of colony-forming assays showed that the miR-7239-3p Inhibitor group displayed and fewer colonies (*P* < 0.05; Fig. 6E, H), indicating weakened cell proliferation. In addition, transwell assays showed a decrease in the number of cells crossing the diaphragm in the miR-7239-3p Inhibitor group (*P* < 0.01; Fig. 6F, I), indicating reduced invasiveness. Unsurprisingly, CCK-8 assays showed that the cell survival rate in the miR-7239-3p inhibitor group was also lower than that in the control group (*P* < 0.001; Fig. 6G). And the percentage wound healing in the miR-7239-3p inhibitor group was lower than that in the control group (*P* < 0.05; Fig. 6J, L).

The RT-qPCR results demonstrated an increase (*P* < 0.001) in the expression of *Bmal1* and decreased expression of miR-7239-3p (*P* < 0.001) in the miR-7239-3p inhibitor group (Fig. 6K), indicating that miR-7239-3p expression was inhibited. Western blot analysis showed that the expression levels of BMAL1 and E-Cadherin were increased in the miR-7239-3p Inhibitor group (*P* < 0.01, *P* < 0.001), while the expression of N-Cadherin and Vimentin were decreased (*P* < 0.01; Fig. 6M, N).

### Negative Correlation Between Bmal1 and miR-7239-3p Expression *In Vivo*

**Fig. 7:**
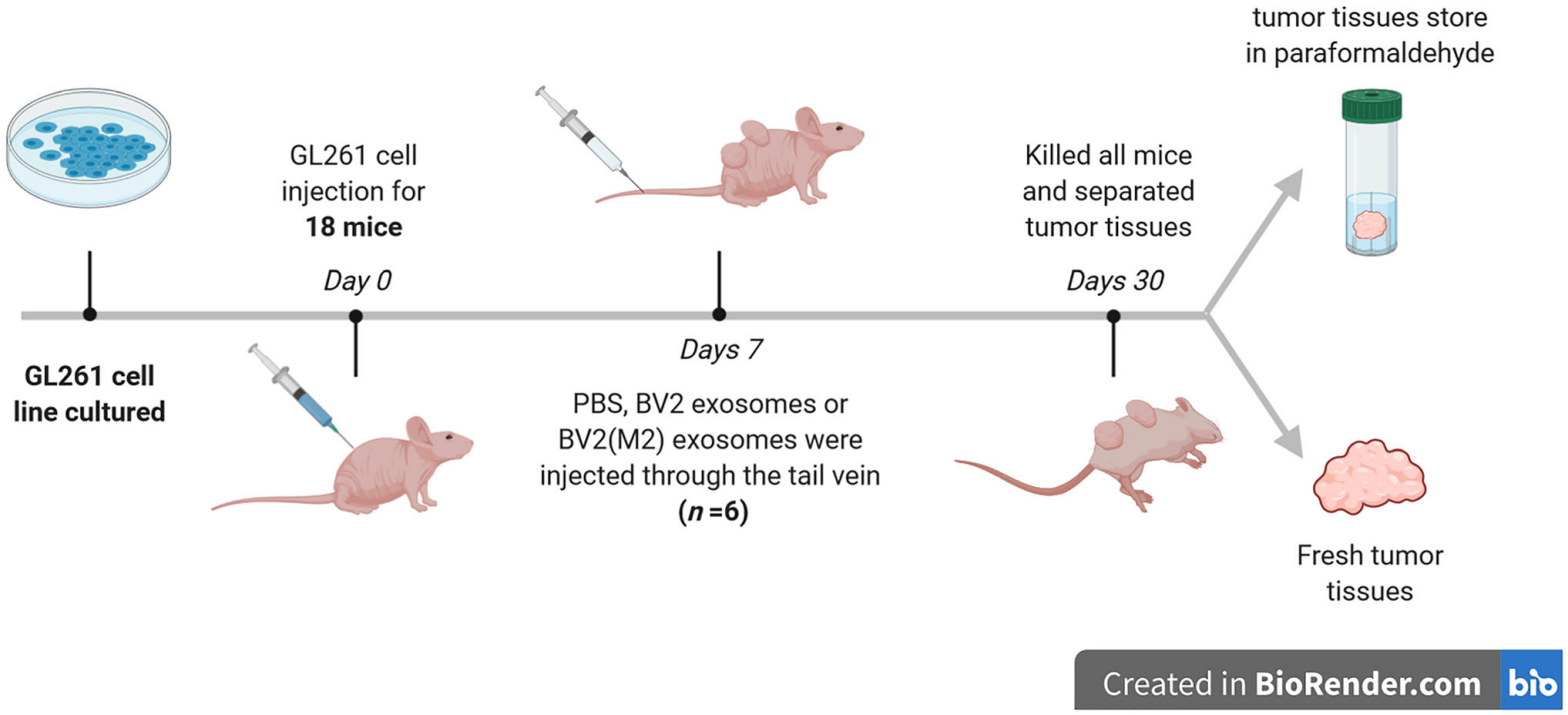
Overview of the *in vivo* experimental procedure.

In order to investigate the role of M2 microglia-derived exosomes *in vivo* and further explore the molecular function of miR-7239-3p in glioma progression, we established a mouse subcutaneous glioma model and injected M2 or unpolarized microglial exosomes through the tail vein. After the mice were sacrificed, the RNA expression levels of *Bmal1* and miR-7239-3p in tumor tissue from each group were measured (Fig. 7). The results showed that the expression of *Bmal1* in tumor tissue from the GL261 + BV2 (M2) Exosomes group was reduced (*P* < 0.001), while the expression of miR-7239-3p was increased (*P* < 0.001; Fig. 7A). This is in contrast to the results from the GL261 group, where *Bmal1* expression was increased (*P* < 0.001) but miR-7239-3p expression was decreased (*P* < 0.001; Fig. 7A). Collectively, the correlation analysis illustrated that the expression level of miR-7239-3p was negatively correlated with that of *Bmal1* (*r* = −0.847, *P* < 0.001; Fig. 7B).

### M2 Phenotype Microglial Exosomes Promote Glioma Growth in vivo

**Fig. 8:**
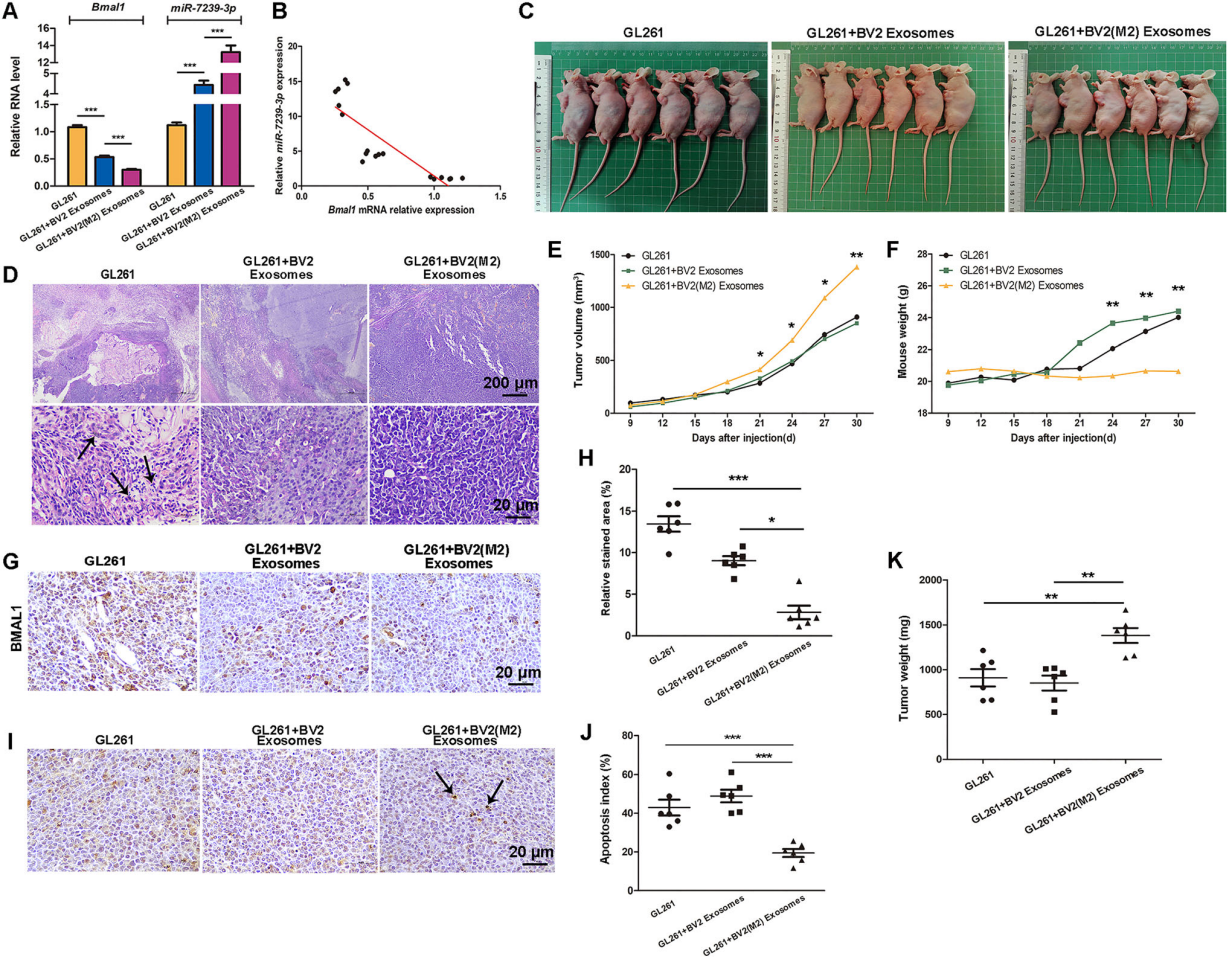
M2 phenotype microglial exosomes promote glioma growth *in vivo*. **A** Real-time qPCR was used to verify relative expression levels of *Bmal1* and miR-7239-3p in tumor tissues. The internal reference genes were *β-actin* and *U6*, respectively. **B** Fitting curve of the correlation analysis between the relative expression levels of miR-7239-3p and *Bmal1*. **C** The subcutaneous tumor formation in nude mice. **D** The pathological morphology of glioma tissue after HE staining. Arrows indicate the fragmented nuclei (karyorrhexis). **E** The curve graph of tumor volume changes with time in each group. **F** The curve of mouse weight change with time in each group. **G, H** IHC was used to detect the expression of BMAL1 protein in each group. **I** The TUNEL apoptosis staining of each group. **J** The statistical analysis results of apoptosis index. **K** The tumor weight in each group (*n* = 6). *P < 0.05, **P < 0.01, ***P < 0.001.

We then explored the expression of BMAL1 protein in tumor tissue using IHC. The results showed that the BMAL1 staining area and intensity in tumor tissue were reduced in the GL261 + BV2 (M2) Exosomes group (Fig. 8G), and by statistical analysis, the expression of BMAL1 protein in this group was reduced (*P* < 0.05, *P* < 0.001; Fig. 8H). This result is in accordance with our findings from RNA quantitation. This evidence further suggested that M2 phenotype microglial exosomes are likely to affect the expression of miR-7239-3p in vivo, which in turn, affects the downstream regulators by down-regulating the expression of BMAL1 protein.

After glioma injection, changes in the tumor volume and mouse weight were recorded every three days. The results showed that the tumor volume increased faster (*P* < 0.05) in the GL261 + BV2 (M2) Exosomes group (Fig. 8E). Intriguingly, mouse weight in the GL261 + BV2 (M2) Exosomes group did not significantly increase throughout the study, while mice in the other two groups displayed a significant increase in weight from the day 24 (*P* < 0.01; Fig. 8F). On the day 30 after glioma injection, we killed the mice, took their pictures (Fig. 8C), and recorded the tumor weight. The results showed that the tumor weight was larger (*P* < 0.01) in the GL261 + BV2 (M2) Exosomes group (Fig. 8E).

We used HE staining to observe the morphological characteristics of tumor cells in each group, and found apoptosis in glioma tissue by TUNEL assays. The results showed that in the GL261 + BV2 (M2) Exosomes group, the tumor cells were closely arranged with a normal size and clear structure. Both the GL261 group and the GL261 + BV2 Exosomes group displayed reduced cell body size and enlarged gaps, and in addition, nuclear shrinkage and fragmentation were observed in some of the cells (Fig. 8I). TUNEL assay results showed that while a large number of apoptotic cells was observed in the GL261 group and GL261 + BV2 Exosomes group, with apoptosis indexes of 42.93 ± 10.07% and 48.90 ± 7.98%, respectively, while the GL261 + BV2 (M2) Exosomes group showed a reduction in apoptotic bodies (*P* < 0.001), with an apoptosis index of 19.43 ± 5.09% (Fig. J).

## Discussion

GAMs occupy the largest proportion of tumor infiltrating cells, accounting for 30% to 70% of the volume of gliomas. The cytokines and chemokines produced by GAMs help tumor growth and maintain the immunosuppressive microenvironment [17]. Lisi *et al*. studied the activation of microglia in clinical samples from 41 patients with grade IV glioblastoma, and found that expression of M1 and M2 microglial polarization markers was tightly correlated with the mean survival times of patients suffering glioblastoma [18]. da Fonseca *et al.* analyzed the tumor center and surrounding parenchyma of each patient, and considered that the M2 microglial markers IBA-1, CD163, iNOS, and ARG-I were prognostic marker, rather than M1 [17]. This indicates that M2 microglial cells play an important role in glioma-mediated immune evasion. Therefore, we chose M2 microglia for the current study. We found that distinctive phenotypes of microglial cells regulate the expression of circadian genes in gliomas: after co-culture with M2 microglia, the expression of BMAL1 protein decreases, while CLOCK expression increases. We confirmed that the M2 microglia promote the proliferation of glioma using CCK-8 assays and FCM, which is consistent with the recent findings by Lisi *et al.* [18].

In our previous work, we have found that the growth of a variety of tumor cells is related to disturbances in circadian rhythms or changes in circadian genes [19, 20]. The *Bmal1* gene is down-regulated in a variety of tumors, which inhibits tumor growth both *in vivo* and *in vitro* [21]. *Clock* gene expression varies with tumor types. For example, *Clock* genes are under-expressed in a variety of malignant tumors such as ovarian, prostate, and pancreatic ductal carcinomas [22, 23]. However, the expression of *Clock* in colorectal cancer and breast cancer is elevated [24]. Therefore, in our study, it seems understandable that the expression of BMAL1 protein and CLOCK protein showed opposite trends. Finally, we used the lentiviral transfection technique to stably down-regulate *Bmal1* in GL261 cells and found that such down-regulation enhanced the proliferation and migration of glioma cells, which in turn, promoted the progress of glioma.

miRNAs have great potential in developing biomarkers for tumor diagnosis and prognosis [25]. Exosomes can protect miRNAs from degradation, stably express them in extracellular space, and deliver them to specific recipient cells [26, 27]. We predicted miR-7239-3p to target the *Bmal1* 3’UTR. After treating glioma cells with M2 and unpolarized microglial exosomes, we found that the expression of *Bmal1* and miR-7239-3p were significantly different in the two groups. Treating glioma cells with M2 microglial exosomes enhanced their growth, and migration, and invasion capabilities. The changes in the expression of E-Cadherin and N-Cadherin proteins are consist with the trend in the malignant progression of most tumor cells. E-Cadherin and N-Cadherin proteins are important during the epithelial-mesenchymal transition (EMT), and the EMT is the initial cause of tumor invasion and metastasis [28]. One of the markers of the EMT is the loss of epithelial cell integrity, accompanied by weakened adhesion between epithelial cells. In this process, the gene transcription of epithelial-specific proteins (such as E-Cadherin) is suppressed by EMT-promoting transcription factors, which promote the degradation of adhesion junctions. At the same time, epithelial cell-specific proteins are replaced by more flexible cadherins (such as N-Cadherin), which promote cell isolation and enhance cell mobility [29]. Our results demonstrate that the Exosomes of M2 microglia down-regulate E-Cadherin and up-regulate N-Cadherin expression, promoting the malignant process of gliomas. In addition, Vimentin negatively regulates E-Cadherin and participate in the process of cell invasion [30], providing a sufficient argument for the above results. After adding a specific inhibitor of miR-7239-3p, the tumor-promoting effect of M2 microglial exosomes was significantly reversed.

Finally, by establishing a glioma model in nude mice, we found that miR-7239-3p and *Bmal1* gene expression were negatively correlated. Therefore, we speculate that exosome miR-7239-3p is an important intermediate molecule in the M2 microglial promotion of glioma progression. There are several limitations of our study that should be recognized. Although the circadian rhythm system is highly conserved in biological evolution, and has a high degree of homology in mice and humans, our findings may not be reflected in all biological settings. Further studies are needed to test our findings in the context of human cell lines and allow the research community to better understand the biology of glioma. In addition, given the presence of the blood-brain barrier (BBB), exosomes injected through the caudal vein may not be able to reach the tumor microenvironment through blood circulation. Although exosomes have reportedly become an attractive vehicle for targeting drugs to enter the brain, whether or how they cross the BBB remains unclear [31]. Therefore, we established the subcutaneous tumor model for exploratory experiments, instead of an orthotopic xenografts of GL261 into the brain of immunocompetent mice. In future studies, we hope to establish a orthotopic xenograft glioma model, and conduct the exosome implantation through local invasive intracerebroventricular administration, infusion, intranasal administration, or induction of permeability by temporary disruption of the BBB (e.g., by ultrasound), and further explore the specific criteria for these exosome implantations.

Our work revealed that miR-7239-3p, which is secreted by M2 microglial exosomes, enters glioma cells by endocytosis, leading to the inhibition of *Bmal1* gene expression, and ultimately the promotion of glioma progression. Inhibiting the secretion of M2 microglial exosomes or prevention of M2 microglia polarization may be new directions for glioma treatment.

## Conclusions

In summary, we found that miR-7239-3p in the glioma microenvironment is recruited to glioma cells by exosomes and inhibits *Bmal1* expression. M2 microglial exosomes promote the proliferation and migration of gliomas by regulating tumor-related protein expression and reducing apoptosis. We discovered the mechanism by which M2 microglia promote glioma progression, and the role of miR-7239-3p-carrying M2 microglial exosomes in the tumor microenvironment, which may provide a theoretical basis for the development of targeted therapies for glioma patients.

## Supplementary Information

Below is the link to the electronic supplementary material.

Supplementary material 1 (PDF 92 kb)
