# Supplementary material for: Microglial Exosome miR-7239-3p Promotes Glioma Progression by Regulating Circadian Genes
Source: Neurosci Bull. 2021 Feb 2;37(4):497–510. doi: 10.1007/s12264-020-00626-z (PMC8055789; doi:10.1007/s12264-020-00626-z)
Supplement: Supplementary file 1 — Supplementary material 1 (PDF 92 kb) [file 12264_2020_626_MOESM1_ESM.pdf]

Supplementary Materials

Table S1. Primers for Quantitative Real-Time PCR

Primer name	Sequence
iNOS-Forward	5’-TTTGGGAATGGAGACTGT-3’
iNOS-Reverse	5’-AAGGTGTGGTTGAGTTCT-3’
IL-12-Forward	5’-CCTTGGTAGCATCTATGA-3’
IL-12-Reverse	5’-GGTTGTGATTCTGAAGTG-3’
Arg-1-Forward	5’-CCAGATGTACCAGGATTC-3’
Arg-1-Reverse	5’-GCTTGTCTACTTCAGTCA-3’
IL-4-Forward	5’-ATGCTTGAAGAAGAACTCTA-3’
IL-4-Reverse	5’-GTGGACTTGGACTCATTC-3’
Bmal1-Forward	5’-TAGCCAGAATGACCTTATTG-3’
Bmal1-Reverse	5’-AGTGTCCGAGGAAGATAG-3’
β-actin-Forward	5’-TATGGAATCCTGTGGCATC-3’
β-actin-Reverse	5’-GTGTTGGCATAGAGGTCTT-3’
U6-Forward	5′-GCGCGT CGTGAAGCGTTC-3′
U6-Reverse	5′-GTGCAGG GTCCGAGGT-3′

#
